# Vulnerability of Thermal Energy Storage Lining Material to Erosion Induced by Particulate Flow in Concentrated Solar Power Tower Systems

**DOI:** 10.3390/ma17071480

**Published:** 2024-03-24

**Authors:** Zeyad Al-Suhaibani, Nader S. Saleh, Shaker Alaqel, Rageh Saeed, Eldwin Djajadiwinata, Syed Noman Danish, Hany Al-Ansary, Abdelrahman El-Leathy, Sheldon Jeter

**Affiliations:** 1 Mechanical Engineering Department, King Saud University, P.O. Box 800, Riyadh 11421, Saudi Arabia; 2 K.A.CARE Energy Research and Innovation Center at Riyadh, King Saud University, P.O. Box 800, Riyadh 11421, Saudi Arabia; 3 Sustainable Energy Technologies Center, King Saud University, P.O. Box 800, Riyadh 11421, Saudi Arabia; 4 Georgia Institute of Technology, School of Mechanical Engineering, 771 Ferst Drive, Atlanta, GA 30332, USA

**Keywords:** thermal energy storage, concentrated solar power, particle-based thermal energy storage bin, solar particle heating system

## Abstract

Researchers from all around the world have been paying close attention to particle-based power tower technologies. On the King Saud University campus in the Kingdom of Saudi Arabia, the first integrated gas turbine–solar particle heating hybrid system has been realized. In this study, two different types of experiments were carried out to examine how susceptible prospective liner materials for thermal energy storage tanks were to erosion. An accelerated direct-impact test with high particulate temperature was the first experiment. A low-velocity mass-flow test was the second experiment, and it closely mimicked the flow circumstances in a real thermal energy storage tank. The tests were conducted on bare insulating fire bricks (IFBs) and IFBs coated with Tuffcrete 47, Matrigun 25 ACX, and Tuffcrete 60 M. The latter three lining materials were high-temperature-resilient materials made by Allied Mineral Products Inc. (AMP) (Columbus, OH, USA). The results showed that although IFBs coated with AMP materials worked well in this test, the accelerated direct-impact test significantly reduced the bulk of the bare IFB. As a result, lining substances must be added to the surface of IFBs to increase their strength and protection because they cannot be used in situations where particles directly impact their surface. On the other hand, the findings of the 60 h cold-particle mass-flow test revealed that the IFBs were not significantly eroded. Additionally, it was discovered that the degree of erosion on the samples of bare IFB was unaffected by the height of the particle bed.

## 1. Introduction

**Figure 1:**
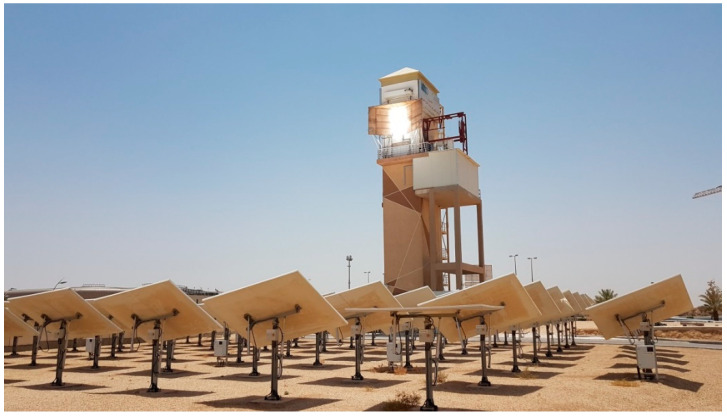
World’s first hybrid PBPT system at King Saud University, Riyadh, Saudi Arabia.

Particle-based power tower (PBPT) systems have been attracting considerable attention from researchers worldwide. PBPT devices can overcome the present constraints of molten salt receivers, where temperatures typically cannot surpass 600 °C because the working and storing medium are particle materials. Numerous applications, including supercritical CO_2_ power cycles [1,2,3,4,5,6,7,8,9,10,11,12], air–Brayton power cycles [13,14,15,16,17,18,19,20,21], and high-temperature process heat applications [22,23,24,25,26], are made possible by the higher temperatures that PBPT systems can reach. Additionally, the cost of thermal energy storage (TES) can drop dramatically, particularly if natural particle materials are employed [27,28,29,30,31]. The studies being conducted by the authors at King Saud University (KSU), Saudi Arabia, for the last fifteen years, involve extensively evaluating the innovative Concentrated Solar Power (CSP) systems, especially PBPT systems, and proving their techno-economic viability [13,14,15,16,17,27,28,29]. The authors designed and completed the first purpose-built PHR system in the world (Figure 1), which is located at Riyadh Techno Valley at KSU. Furthermore, the authors are uniquely active in all aspects of PHR technology including the development of receiver, heat exchanger, particulate materials, and particulate handling technologies.

One of the important performance metrics that needs to be met is to keep the cost of the TES system below USD 15/kWh (thermal) [32,33]. The two main budget components of the TES subsystem are the particulate material itself and the containment/bin construction; therefore, it is important to find ways to minimize the cost of both components. At Sandia National Laboratories (SNL), Martin and Vitko [34] studied the applicability of storing thermal energy in solid particles. In the early 1980s, an effort was invested to conceptualize a direct-absorption central receiver. The targeted system was aimed to produce electric power with a potential implementation for chemical production cycles at high temperatures (>600 °C). Later, additional effort was put into examining the use of solid particles to serve as a storage and working medium [35]. Falcone et al. [36,37] developed a methodology that can be used for a particulate selection and receiver design. An inexpensive alumina-based particulate material, which can work at temperatures of up to 1000 °C, was introduced as a potential candidate for use as a storage and working medium.

At King Saud University (KSU), several particulate candidates have been proposed and tested. Those candidates (including CARBOBEAD CP (USD 2.2 per kg), red sand, and white sand (USD 0.13 per kg) vary greatly in cost and performance [38]. In order to investigate the response of particles, samples were heated repetitively in an electric oven at 1200 °C for multi-six-hour cycles to achieve a total of 500 h of testing [39]. Results showed that red sand exhibit agglomeration and color change. Red sand particles formed a bigger lump, and the reddish color changed to white with time, and the packed-bed solar absorptance decreased significantly. On the other hand, white sand showed no agglomeration tendency after cyclic heating for 500 h. As for CARBOBEAD CP particles, soft lumps or loose agglomerations were formed which easily disappeared when pouring the particles out of the testing crucible.

Similarly, researchers worldwide are seeking a simple, efficient, and inexpensive containment structure. Zunft et al. [40] studied and calculated the heat losses from a rectangular storage bin. Four parallel compartments holding ceramic storage material made up the test equipment. The results showed that at a core temperature of 630 °C, 55% of the heat loss was attributed to losses through the lateral sides of the bin. Several TES systems were studied and evaluated by Nandi et al. [41,42,43]. Heat losses and material cost were the main metrics of the study, which included a thermocline, phase change material (PCM), two-tank system, castable ceramic, and concrete. The storage system is rated to a solar power plant of 50 MW with a storage duration of 6 h. The results showed that the thermocline system was the most cost-effective and efficient of all systems. Recently, Zhao and a team [44,45] examined gas–solid thermochemical energy storage and demonstrated that, because of its high energy storage density and effective power generation at a high discharge temperature, this technology is promising for storing and using renewable energy, such as CSP, and excess electricity from all types of renewables.

At KSU, a multi-layered TES bin design was thoroughly examined. Alumina-rich Insulating Fire Brick (IFB), perlite concrete with refractory cement, an expansion board, and reinforced concrete make up the four layers of this construction, which are arranged from the inner to the outer parts [46]. It has been estimated that the cost of the containment structure at a large scale is on the order of USD 2.6/kWh_th_, which is very low. However, one of the major concerns about this design is the fact that the IFB is brittle by its nature. With the continuous flow of particles against the IFB layer, erosion can take place over time. If the mass loss is significant, it will lead to two major issues. First, the thermal performance of the containment structure will be gradually degraded. Second, the eroded layer will mix with the particulate TES material, thereby changing its composition and potentially creating “nucleation” sites for sintering.

The literature review reveals that a lot of research has been conducted by researchers around the world. However, the authors at KSU are pioneers in developing a unique concept of particle-based CSP, and the research presented in this paper has never been investigated, especially the erosion induced by high-temperature particulate flow. This study was prompted by an observation of blockages occurring at the outlet opening of the heat exchanger during on-sun tests conducted at the 300 kW thermal particle-based CSP system located on the campus of King Saud University, Riyadh, Saudi Arabia. The blockages were found to be caused by broken IFBs originating from the thermal energy storage bin positioned above the heat exchanger. Therefore, this experimental investigation aimed to study the susceptibility of IFB and other potential lining materials to erosion due to particle flow. The main criteria of material selection were a high abrasion resistance and a capability to withstand high temperatures. Since the TES medium is made of solid particles, it is required to avoid contaminating the particles with the eroded layer. This would lead to changing the TES medium’s composition and potentially creating nucleation sites for sintering. The investigated lining materials include three different materials produced by Allied Mineral Products (AMP) Company, namely, Matrigun 25 ACX, Tuffcrete 47, and Tuffcrete 60 M.

## 2. Experimental Setup and Tested Materials

There were two distinct kinds of experimental runs. The first type was a high-temperature, accelerated direct-impact test, and the second was a low-velocity mass-flow test that closely mimics the flow patterns in a real TES tank or bin. The following gives a detailed description of the two experimental setups.

### 2.1. Accelerated High-Temperature Direct-Impact Experiment

The goal of this test was to determine whether the refractory materials could withstand the force that they would potentially experience when filling the TES bin. The test was run using KSU’s current PBPT system. It includes a heliostat field, a particle heating receiver (PHR), a TES tank, a particle-to-working fluid heat exchanger (PWFHX), a power block (microturbine), and a particle circulation mechanism, all of which are vertically stacked. A particle lift raises particles to the top of the tower, where they are then released at the PHR to directly intercept and absorb the concentrated sunlight that is reflected. The particles are then sent to the TES tank, which feeds the PWFHX, which later transfers the energy to the air used in the power cycle. The particle lift then returns the cooled particles to the PHR, completing the particle loop. In [9,10,11,12,13,14], the operation of the plant is discussed in more detail.

**Figure 2:**
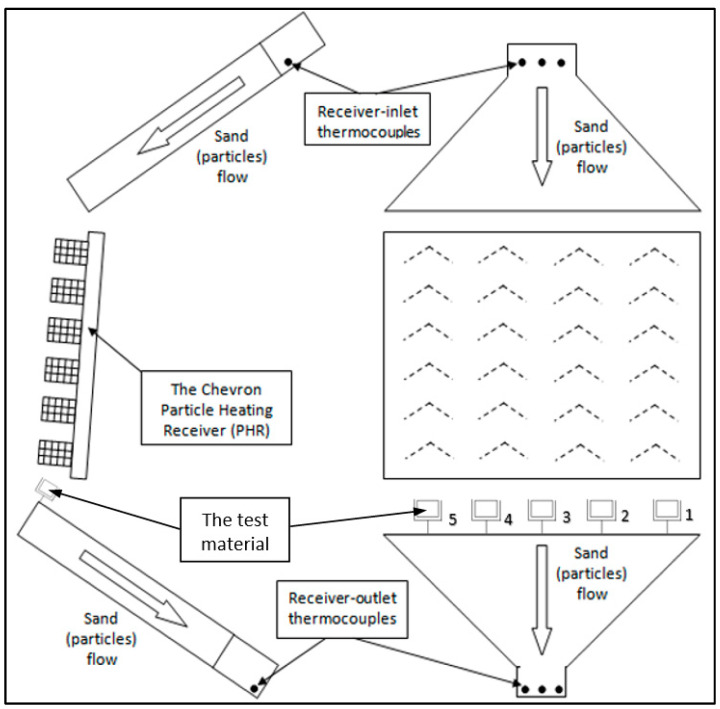
A schematic representation of the direct-impact test apparatus.

At the PHR discharge, i.e., directly under the PHR, a test rig was constructed and set up. Five samples of the tested materials can be accommodated on the rig at once. Additionally, each mounting of the sample was made to be able to slide or rotate, allowing the samples to be positioned and aligned as needed. Figure 2 depicts the schematic of the test rig.

Riyadh red sand was the particle material used in this test. The decision to use red sand in this study does not contradict the conclusions presented by the authors in a previous study [39]. Specifically, the current tests were conducted at temperatures below the threshold at which red sand begins to agglomerate, which is approximately 600 °C. It is important to note that red sand shares similar characteristics with white sand, except for its color and tendency to agglomerate. Therefore, the utilization of red sand as the working media aligns with the main objective of the study, allowing for a comprehensive investigation into particle abrasion on the lining material. To account for measurement variability, each of the five samples was weighed at least 6 times both before and after the test. An accurate weighing scale (PLJ4000-2M, KERN) [47] was used for mass measurement. The scale has an accuracy of ±0.1 g. After every 12 h, each sample was weighed six times using this scale; this was meant to reduce the uncertainty of the measurement. The random uncertainty was calculated based on the Kline and McClintock method [48]; as an example, the measured masses for samples 1 and 12 are listed in Table 1.

The velocity of the particles was calculated based on the measurements of the flow rate and the cross-sectional area of the test setup. The flow rate measurement was conducted using simple weight and time measurements. The superficial velocity of particles was calculated using the following equation:

(1) $$V_{p}=\frac{{\dot{m}}_{p}}{\rho_{p}A_{cs}}$$

where $V_{p}$ is the particle’s superficial velocity, $\rho_{p}$ is the material density of particles, and $A_{cs}$ is the cross-sectional area of the test setup.

The mass flow rate ${\dot{m}}_{p}$ was calculated as

(2) $${\dot{m}}_{p}=\frac{\Delta m}{\Delta t}$$

**Figure 3:**
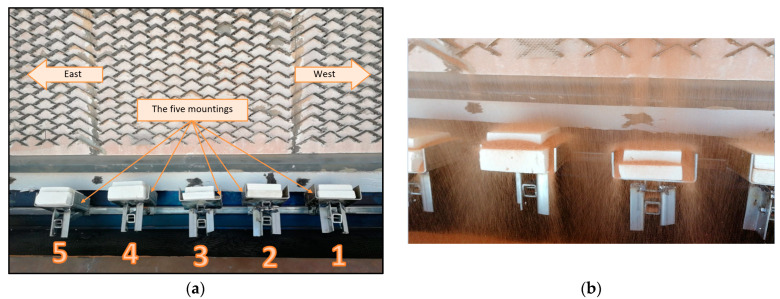
(**a**) In the test rig shown in the image, the five mountings can slide and rotate in the west-to-east direction; (**b**) the tested samples (bare IFB samples) during the hot-particle impact test.

The test was carried out in two stages, with the first stage employing solely bare or liner-free IFBs. The particles were arranged at an angle greater than the red sand angle of repose (30° from the horizontal line) to guarantee that they flowed upon impact on the IFB samples. In Figure 3 of the test rig, five samples of IFBs are displayed.

**Figure 4:**
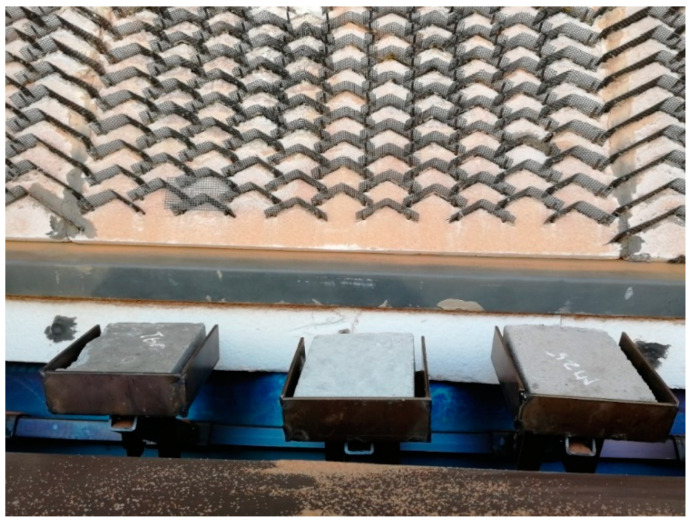
The three samples of IFB coated with AMP lining materials (Tuffcrete 60 M, Tuffcrete 47, and Matrigun 25 ACX, from left to right) and the direct-impact test equipment.

In the second stage, the IFB coated with three lining materials was tested. These lining materials were manufactured by AMP. They are called (1) Matrigun 25 ACX [49], (2) Tuffcrete 47 [50], and (3) Tuffcrete 60 M [51]. Their maximum operating temperature was 1300 °C, 1540 °C, and 1700 °C, and their densities were 1.95, 2.21, and 2.53 g/cm^3^, respectively. The chemical compositions of these materials along with the IFB are listed in Table 2. A brief description of AMP materials along with the installation method is tabulated in Table 3. The test samples before starting the test can be seen in Figure 4.

The test was conducted using red sand particles with a temperature of ~500 °C. The particles were brought to such a high temperature by using the recuperated compressed air supplied by the microturbine of the PBPT system during the night. The particles were kept circulated inside the system. As they moved through the PWFHX, they were heated. The particulates were permitted to flow through the PHR on test day and were then heated more by the intense sunlight converged by the 66 heliostats.

### 2.2. Low-Velocity Cold Experiment

**Figure 5:**
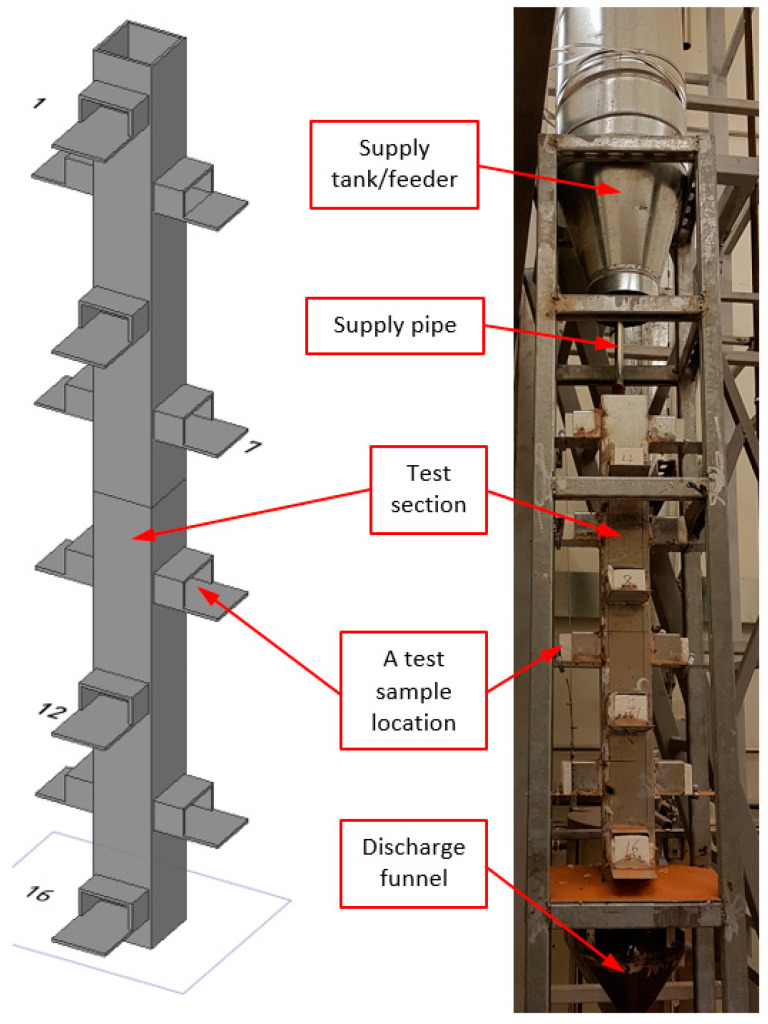
The cold-particle mass flow test rig (1 m high).

The flow characteristics in a real TES tank were precisely replicated in this test. The test unit was a 1 m high rectangular steel sheet duct with sixteen rectangular perforations on each side. As seen in Figure 5, these holes were utilized to house the test samples. There were eight separate levels, going from top to bottom, and each had two test samples installed (for a total of sixteen samples). The cross-sectional area of the test section through which the particles flowed was 100 mm by 80 mm. Each test sample had a test surface that was around 60 mm by 56 mm in size, and the particulate flow was parallel to the surface of the test rig.

Before placing them at their positions, the weights of the sixteen samples of the tested bare IFB were measured. The test section was then completely packed with the particulates after the test samples had been positioned in their predetermined locations. The pipe end of the discharge funnel was now covered by a cap. No valves were employed in this test apparatus. The supply pipe aperture was made larger than the discharge pipe opening by using different pipe sizes, i.e., 12-inch and 34-inch pipes for the discharge and the supply, respectively, to constantly have the test section full of particles. Additionally, the supply pipe (Figure 5) was extended in the direction of the top aperture of the test section to stop the particles from overflowing it. Every time the test section was full, the accumulation of particulates would obstruct the supply pipe, and the supply flow would stop. To ensure that there was no pollution in the tank or the test area, a filter was installed at the supply tank (feeder) inlet. The discharge funnel pipe cover was removed when the test section was fully loaded, and the particles were then allowed to pass through for a total of about 60 h while being monitored every 12 h. To conduct the assessment, all of the test samples were taken out of the test section, and then their weights were measured. To determine whether there had been any mass loss, the current weight was compared with the prior weight.

## 3. Results

### 3.1. Accelerated High-Temperature Direct-Impact Experiment

**Figure 6:**
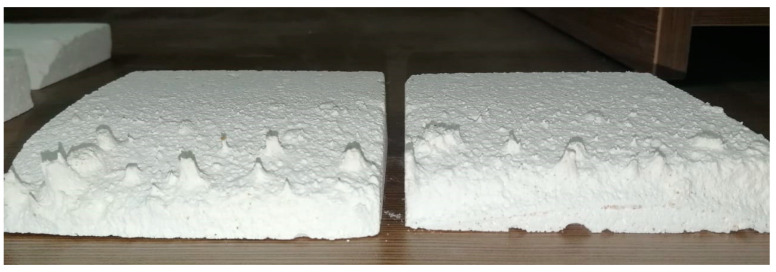
Pitting and deterioration of bare IFB after direct-impact test.

In the first stage of this test, five samples of bare IFB were tested. The samples were placed in the test section at the outlet of the PHR where they were subjected to the direct impingement and flow of falling red sand particles. The particles were at around 500 °C as they were preheated by the working fluid of the power block during the preceding night and heated further by the heliostat field during the following testing day. The test lasted for around one hour. The findings demonstrated that the samples of bare IFB were severely damaged and pitted by the particles falling from the PHR. Two samples of bare IFB following the test are shown in Figure 6. Table 4 displays the sample weights before and after the test. It is obvious that the samples had a significant mass loss in a short amount of time. In circumstances where particles are likely to impinge on its surface, the bare IFB cannot be employed.

A slight mass loss of 1.5 g, 2.0 g, and 21.5 g for the IFB coated with Tuffcrete 60 M, Tuffcrete 47, and Matrigun 25 ACX, respectively, was seen for the test samples in the second stage of the test (Table 5). This is encouraging in comparison to the results of bare IFBs. It must be mentioned that the surface area of the sample was the same size, measuring 100 mm × 114 mm. Because, theoretically, the dimensions of the surface area would be proportional to the quantity of mass eroded, this was performed to compare mass loss fairly.

In an assessment of the findings of the IFB test, the results were positive. As a result, coating the IFBs with one of the AMP lining materials will give the TES tank wall good thermal resistance as well as strength. These lining materials were shown to be excellent candidates to protect the IFBs from erosion, particularly the Tuffcrete 60 M and Tuffcrete 47, whereas the most noticeably deteriorated was the Matrigun 25 ACX.

### 3.2. Low-Velocity Cold Experiment

This test was carried out at a room temperature for 60 h. By dividing the mass flow rate of the particulates by the cross-sectional area of the test section and the particulate density, the surface particulate speed inside the test section was determined to be 3 mm/s. After 60 h of testing, the data revealed that none of the 16 samples of the bare IFBs had seen any appreciable degradation. Additionally, it was discovered that the extent of erosive damage to the samples was unaffected by the height of the particle bed. What follows is an explanation of these conclusions. Table 6 contains all of the test findings. The definition of “Δm_i” in this table is the change between “after the run i mass” and “initial mass”. Thus, the negative sign denotes mass reduction, and the positive sign denotes the opposite.

**Figure 7:**
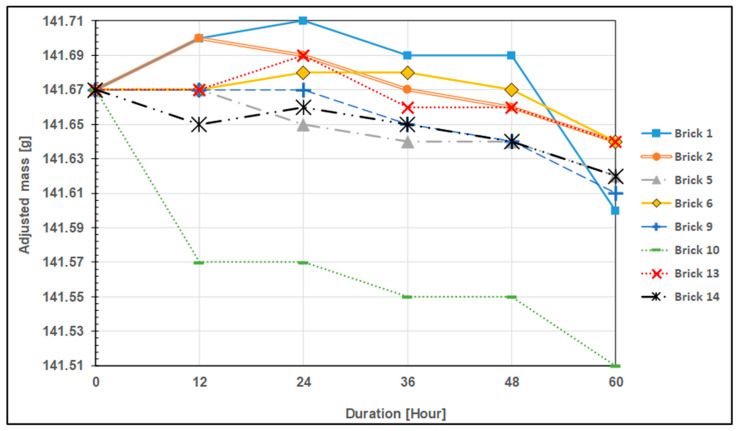
The corrected mass of bare IFB test samples for levels 1, 3, 5, and 7.

**Figure 8:**
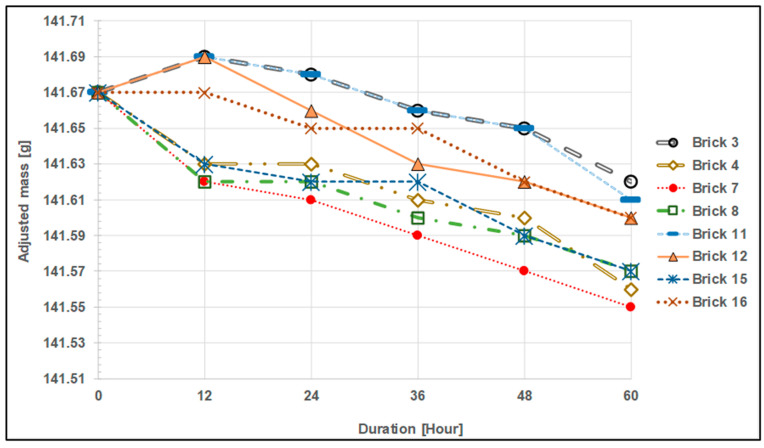
The corrected mass of test samples (bare IFB) for levels 2, 4, 6, and 8.

Some curves depicting the corrected mass of each sample are shown (Figure 7 and Figure 8) to help in comparing the trends of the mass change of the samples. The so-called corrected mass was created by slightly offsetting the masses of each sample, bringing their starting masses into balance. As a reference, the sample with the largest initial mass is picked. It is easier to compare mass change trends between samples in relation to the test period by doing this (offsetting the mass). Brick number 14 has the highest initial mass at 141.67 g, serving as the reference for illustration. An offset value must be applied to the mass data of brick no. 15 in order to determine the corrected mass. Finding the variation between the original masses of brick number 14 (the reference) and brick number 15 yields an offset value of 13.31 g. Table 7 provides a visual representation of this.

**Figure 9:**
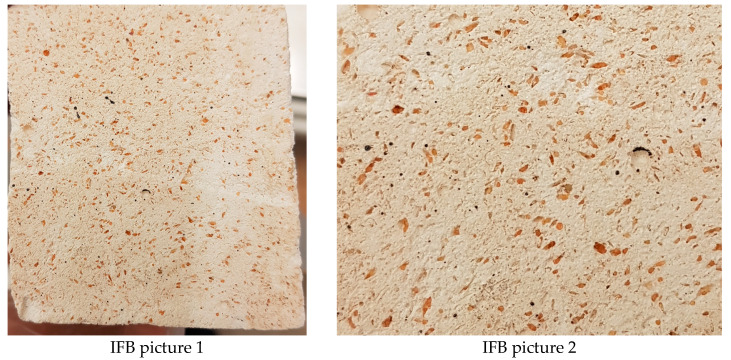
Red sand particles were caught in the surface pores of bare IFB samples (pictures from two different IFBs).

Figure 7 and Figure 8 show that the mass drop trend is a bit irregular, particularly during the first run (12 h), and even through the second run (24 h). This can be explained by two factors. The first is that certain particles were caught in the test surface pores made of IFBs (Figure 9). The bulk grew as a result of this. The second factor is that certain IFB surfaces can start to deteriorate more quickly. This is due to the fact that the samples were made by first cutting them and then smoothening them afterward. As a result of the intrinsic fragility of the IFB, there is a potential that a tiny, invisible crack will form on the test surface during preparation. After the initial run, this tiny crack disintegrates, resulting in a bigger loss of mass. Few test outcomes that show no mass loss may be the result of a combination of mass loss from existing erosion and mass gain from the particulates accumulated on the test surface. They completely negate one another.

Nevertheless, despite the previously noted unpredictability, it is generally evident that many of the mass reduction rates of the samples after the 12 h test are comparable, as shown by their slope in Figure 7 and Figure 8. These statistics and Table 8 below show that this pattern is much more obvious after the 24 h test. Based on the results of the test from the 24 h test to the 60 h test, the table displays the slope of the trend-line for each sample (aside from sample No. 1) as well as the rate of mass drop per unit area. Sample No. 1 is not included in this table since the 60 h test result is inaccurate due to the improper handling that occurred during the evaluation. It must be emphasized that because erosion is a surface-level phenomenon, the area size will be inversely proportional to the rate of mass loss. As a result, it makes more sense to express the rate of mass loss in the form of per unit test area. With a precision error (95% confidence level) of 0.047 g/(h·m^2^), or around 11% of the average value, it is determined that the average mass loss rate is 0.425 g/(h·m^2^). Reiterating, the test section height (the height of the particle column) was 1 m, and the particle’s superficial speed was 3 mm/s.

Finally, the results indicate that there is no correlation between the height of the particle bed or column and the rate of erosion of the test samples. Table 8 and the previously provided Figure 7 and Figure 8 make it abundantly evident that many of the samples had comparable rates of mass loss per unit area. This finding might not hold true for a particle bed or column that is significantly larger, such as the one found in the TES tank of large-scale particle CSP systems.

It is worth mentioning that since we did not find any significant erosion of the bare IFB in the low-velocity cold experiment, there was no need to repeat the same test for the coated IFB, which was already strengthened by the AMP lining material that had passed the more rigorous accelerated high-temperature direct-impact experiment.

## 4. Conclusions

Tests were conducted by using the existing particle-based power tower system at KSU. Riyadh red sand was the particulate material utilized during these tests. Accelerated direct-impact testing and low-velocity mass flow testing were both conducted. At the PHR outflow, or directly under the PHR, a test rig holding the materials for the tested TES tank was constructed. The results showed that even though IFBs coated with AMP materials succeeded in this test, the accelerated direct-impact test significantly reduced the bulk of the bare IFB. The surface of the IFBs must be lined with a material to increase protection and strength, particularly where particles may impinge on it. For the purpose of simulating particulate flow inside TES tanks, a steel sheet test rig with a rectangular cross-section and a height of 1 m was built. On its sides, sixteen holes were drilled so that the IFB samples could be inserted. Throughout the test period, the particle flow pattern remained a packed-bed, and the particles’ superficial speed was determined to be 3 mm/s. The primary finding was that at 0.425 g/(h·m^2^), the rate of mass loss per unit surface area remained essentially constant. Even though this rate seems low, in a long-term operation, the mass loss can be significant, which can also cause the particle material to be significantly contaminated.

## Figures and Tables

**Table 1:** Uncertainty in measurement of mass.

Sample Number	Measured Mass (g)	Random Uncertainty
1	133.43, 133.43, 133.43, 133.43, 133.42, 133.42	±0.004
12	135.11, 135.11, 135.11, 135.11, 135.12, 135.11,	±0.003

**Table 2:** The chemical compositions of IFB and AMP lining materials.

	Al_2_O_3_	SiO_2_	CaO	Fe_2_O_3_	TiO_2_	Alkalis	Others
Matrigun 25 ACX	43.9%	40.8%	8.6%	2.4%	-	-	4.3%
Tuffcrete 47	47.1%	46.5%	2.1%	0.8%	2.0%	1.1%	0.1%
Tuffcrete 60 M	60.5%	34.3%	1.9%	0.9%	1.9%	0.2%	0.3%
IFB [52]	37.0% Min.	46.0% Max.	15.2	0.9% Max.	0.5%	1.9% Max.	-

**Table 3:** Description and installation method of AMP lining materials.

Product Name	Description	Installation Method
M Matrigun 25 ACX	General purpose, non-wetting to aluminum, super duty gunning mix	Vibration, pumping, shotcrete, self-flow, gunning, hand packing
Tuffcrete 47	Fire clay-based low-cement mix	Vibration, pumping, shotcrete, self-flow
Tuffcrete 60 M	Alumina-based, low-cement, high-purity castable	Vibration, pumping, shotcrete, self-flow

**Table 4:** Mass loss in the accelerated high-temperature direct-impact test of bare IFB.

Sample	Initial Mass (±0.01g)	Mass after Test (±0.01g)	Loss (±0.01g)	Loss (%)
1	84.78	77.83	06.95	08.19
2	96.21	82.85	13.36	13.89
3	77.71	67.03	10.68	13.74
4	82.57	74.78	07.79	09.43
5	84.49	66.60	17.89	21.18

**Table 5:** Mas loss during the accelerated high-temperature direct-impact experiment performed on the IFB coated with AMP lining materials.

IFB Coated with	Initial Mass [±0.01 g]	Mass after Test [±0.01 g]	Mass Loss [±0.01 g]
Matrigun 25 ACX	708.47	686.93	21.54
Tuffcrete 47	827.07	825.06	02.01
Tuffcrete 60 M	803.54	802.07	01.47

**Table 6:** Results of the test on bare IFBs using cold particulate flow erosion.

Brick no.	Initial Mass, 0 h [g]	After Run 1, 12 h [g]	Δm_1 [g]	After Run 2, 24 h [g]	Δm_2 [g]	After Run 3, 36 h [g]	Δm_3 [g]	After Run 4, 48 h [g]	Δm_4 [g]	After Run 5, 60 h [g]	Δm_5 [g]
1	133.43	133.46	0.03	133.47	0.04	133.45	0.02	133.45	0.02	133.36	−0.07
2	125.52	125.55	0.03	125.54	0.02	125.52	0.00	125.51	−0.01	125.49	−0.03
3	139.42	139.44	0.02	139.43	0.01	139.41	−0.01	139.40	−0.02	139.37	−0.05
4	132.88	132.84	−0.04	132.84	−0.04	132.82	−0.06	132.81	−0.07	132.77	−0.11
5	131.74	131.74	0.00	131.72	−0.02	131.71	−0.03	131.71	−0.03	131.69	−0.05
6	131.06	131.06	0.00	131.07	0.01	131.07	0.01	131.06	0.00	131.03	−0.03
7	127.04	126.99	−0.05	126.98	−0.06	126.96	−0.08	126.94	−0.10	126.92	−0.12
8	116.77	116.72	−0.05	116.72	−0.05	116.70	−0.07	116.69	−0.08	116.67	−0.10
9	128.76	128.76	0.00	128.76	0.00	128.74	−0.02	128.73	−0.03	128.70	−0.06
10	139.11	139.01	−0.10	139.01	−0.10	138.99	−0.12	138.99	−0.12	138.95	−0.16
11	135.40	135.42	0.02	135.41	0.01	135.39	−0.01	135.38	−0.02	135.34	−0.06
12	135.11	135.13	0.02	135.10	−0.01	135.07	−0.04	135.06	−0.05	135.04	−0.07
13	127.57	127.57	0.00	127.59	0.02	127.56	−0.01	127.56	−0.01	127.54	−0.03
14	141.67	141.65	−0.02	141.66	−0.01	141.65	−0.02	141.64	−0.03	141.62	−0.05
15	128.36	128.32	−0.04	128.31	−0.05	128.31	−0.05	128.28	−0.08	128.26	−0.10
16	123.37	123.37	0.00	123.35	−0.02	123.35	−0.02	123.32	−0.05	123.30	−0.07

**Table 7:** An illustration of the meaning of corrected mass.

Mass	Duration of Experiment [h]					
	0	12	24	36	48	60
Brick 14 [g]	141.7	141.7	141.7	141.7	141.6	141.6
Brick 15 [g]	128.4	128.3	128.3	128.3	128.3	128.3
Offset Mass-Brick 15 [g]	13.3					
Corrected Mass-Brick 15 [g]	141.7	141.6	141.6	141.6	141.6	141.6

**Table 8:** The slope of each sample trend line (aside from sample No. 1) and the rate of mass loss per unit area.

Brick #	Surface Area of Tested Brick [m^2^]	Slope [g/h]	Rate of Mass Loss per Unit Area [g/h·m^2^]
2	0.00336	−0.0013	0.387
3		−0.0016	0.476
4		−0.0018	0.536
5		−0.0008	0.238
6		−0.0011	0.327
7		−0.0017	0.506
8		−0.0013	0.387
9		−0.0016	0.476
10		−0.0015	0.446
11		−0.0018	0.536
12		−0.0016	0.476
13		−0.0012	0.357
14		−0.0011	0.327
15		−0.0015	0.446
16		−0.0015	0.446
		Average	0.425
		Uncertainty	0.047

## Data Availability

Data are contained within the article.
